# Ensemble Tree-Based Approach towards Flexural Strength Prediction of FRP Reinforced Concrete Beams

**DOI:** 10.3390/polym14071303

**Published:** 2022-03-23

**Authors:** Muhammad Nasir Amin, Mudassir Iqbal, Kaffayatullah Khan, Muhammad Ghulam Qadir, Faisal I. Shalabi, Arshad Jamal

**Affiliations:** 1 Department of Civil and Environmental Engineering, College of Engineering, King Faisal University, Al-Ahsa 31982, Saudi Arabia; kkhan@kfu.edu.sa (K.K.); fshalabi@kfu.edu.sa (F.I.S.); 2 State Key Laboratory of Ocean Engineering, Shanghai Key Laboratory for Digital Maintenance of Buildings and Infrastructure, School of Naval Architecture, Ocean & Civil Engineering, Shanghai Jiao Tong University, Shanghai 200240, China; mudassiriqbal29@sjtu.edu.cn; 3 Department of Civil Engineering, University of Engineering and Technology Peshawar, Peshawar 25210, Pakistan; 4 Department of Environmental Sciences, Abbottabad Campus, COMSATS University Islamabad, Abbottabad 22060, Pakistan; hashir785@gmail.com; 5 Department of Civil and Environmental Engineering, King Fahd University of Petroleum & Minerals, KFUPM BOX 5055, Dhahran 31261, Saudi Arabia; arshad.jamal@kfupm.edu.sa

**Keywords:** FRP, flexural strength, AI, ensemble models, decision tree, gradient boosting tree

## Abstract

Due to rise in infrastructure development and demand for seawater and sea sand concrete, fiber-reinforced polymer (FRP) rebars are widely used in the construction industry. Flexural strength is an important component of reinforced concrete structural design. Therefore, this research focuses on estimating the flexural capacity of FRP-reinforced concrete beams using novel artificial intelligence (AI) decision tree (DT) and gradient boosting tree (GBT) approaches. For this purpose, six input parameters, namely the area of bottom flexural reinforcement, depth of the beam, width of the beam, concrete compressive strength, the elastic modulus of FRP rebar, and the tensile strength of rebar at failure, are considered to predict the moment bearing capacity of the beam under bending loads. The models were trained using 60% of the database and were validated first-hand on the remaining 40% database employing the correlation coefficient (R), error indices namely, mean absolute error, root mean square error (MAE, RMSE) and slope of the regression line between observed and predicted results. The developed models were further validated using sensitivity and parametric analysis. Both models revealed comparable performance; however, based on the comparison of the slope of the validation data (0.83 for GBT model against 0.75 for the DT model) and higher R for the validation phase in case of the GBT model in comparison to the DT, the GBT model can be considered more accurate and robust. The sensitivity analysis yielded depth of the beam as the most influential parameter in contributing flexural strength of the beam, followed by the area of flexural reinforcement. The developed GBT model surpasses the existing gene expression programming (GEP) model in terms of accuracy; however, the current American Concrete Institute (ACI) model equations are more reliable than AI models in predicting the flexural strength of FRP-reinforced concrete beams.

## 1. Introduction

Cement-based materials and concrete are widely used worldwide due to their low porosity and high mechanical strength, taking additional advantage of reinforced steel bars in reinforced concrete (RC) structures without modifying the cementitious properties of the matrix [1]. Various structural facilities such as dams, bridges and nuclear powerplants are constructed from RC due to its high durability and protective nature of concrete for the rebars [2]. Despite the good durability of RC structures, steel bars are susceptible to corrosion [3]; an electrochemical process involving cathodic reduction of oxygen and anodic dissolution of iron in the presence of concrete pore solution acting as an electrolyte [4]; consequently leading to the failure of RC structures [5]. The corrosion mechanism encompasses the infiltration of chloride ions in the porous concrete into the rebar surface under an aggressive environment, degrading the protective layer formed on the steel surface. After steel corrosion, cracks appear on the surface of concrete due to resulting precipitates and the generation of tensile stresses [6]. This leads to frequent maintenance of RC structures, drastically impacting the economy in general [7,8].

The rapid increase in the population of the world poses a huge demand for the development of infrastructure; thus, the production of concrete is considerably increased [9]. The use of fresh water and river sand poses a negative impact on drinking water and river ecosystems. Besides, the coastal infrastructure demands direct use of seawater and sea sand in the concrete to avoid wasting time and money [10]. Concrete made of seawater and sea sand is highly aggressive due to its high alkalinity and chloride ions [11]. The use of conventional steel reinforcement is hindered in such a situation. Fiber-reinforced polymers (FRP) bars offer suitable applications instead of steel reinforcement due to their corrosion resistance [12,13,14,15]. FRP rebars provide several advantages such as high strength to weight ratio, low density, ease of handling, and corrosion resistance; therefore they have been used as internal reinforcement in concrete structures [16,17].

Several types of FRPs rebars, namely carbon FRPs, glass FRPs, aramid FRPs, and basalt FRPs are used as an alternative to conventional steel. Over the past three decades, numerous experimental works have been undertaken to assess the durability of FRPs [18,19,20,21,22,23,24]. The durability of FRPs in normal concrete is very satisfactory; however, its durability in harsh alkaline concrete environments is still debatable [15]. The design of flexural members such as beams and columns are key parameters to structural integrity. American Concrete Institute (ACI) 440.1 R-15 recommends basic formulations for the design of beams on the basis of mechanics [25]. However, Murad et al. [26] found that the flexural strength achieved from FRP reinforced concrete beams has some deviations from experimental test results. Gene expression programming (GEP), a modern Artificial intelligence (AI) technique based on genetic algorithms, can accurately predict the flexural strength of FRPs.

In the modern era, Finite Element Analysis [27,28,29] and AI techniques are widely used for solutions of problems in civil engineering, owing to their high accuracy and efficiency [30,31,32,33,34,35,36,37,38]. Awoyera et al. [39] employed an Artificial Neural Network (ANN) and GEP for estimating compressive, split tensile and flexural strength of geo-polymer concrete. A reliable accuracy of low error indices and high correlation was achieved in predicting strength characteristics of geo-polymer concrete. Wang, X.-Y. [40] successfully estimated the flexural strength of pozzolana and limestone blended concrete deploying ANN and GEP. Ahmad et al. [41] investigated decision tree (DT), ANN and gradient boost (GB) to predict the compressive strength of concrete at high temperature. Aslam et al. [37] employed GEP for the compressive strength of concrete containing Rice Husk Ash (HRA). The strength prediction of FRP-reinforced concrete has been investigated by several researchers using advanced AI techniques. Congro et al. [42] developed an ANN model for estimating the flexural strength of fiber reinforced concrete under bending loads. Lee, S. and C. Lee [43] investigated the ANN model to estimate the shear strength of FRP flexural members without stirrups and found that the ANN model can more accurately estimate the shear strength of FRP reinforced concrete beams compared to existing equations. The application of various AI techniques for predicting the strength characterization of FRP-reinforced and retrofitted concrete can be found in previous research [44,45,46,47].

Decision tree (DT) and Gradient boosting tree (GBT) are modern operational techniques used for regression and classification problems [48]. These techniques develop a tree-like structure in decision making, splitting the data into root node, branch nodes and several lead nodes [49]. Kermain et al. [50] found the GEP model better in predicting dam air demand than GBT and Random Forest (RF). Similarly, Song et al. [51] also found GEP had better accuracy than ANN and DT in predicting compressive strength of fly ash containing concrete. On the other side, Kamari et al. [52] developed GEP, ANN and DT models for characterizing CO_2_-brine solution interfacial tension and found DT more promising compared to GEP and ANN. Ahmad et al. [41] found the GBT model to be more accurate model than the DT or ANN model while investigating the compressive strength of concrete at high temperatures. Similarly, Huat et al. [53] proved GBT a more promising techniques in estimating pile friction-bearing capacity than RF and DT models.

In conclusion, empirical equations are available for the calculation of the flexural capacity of FRP-reinforced beams. Murad et al. [26] investigated the capability of the GEP model and inferred that the developed model is capable of estimating the flexural capacity more accurately. After critically evaluating the results of the GEP model (Section 3.4), it was concluded that Murad et al. [26] based their evaluation on correlation coefficient (R) only; however, error analysis showed that the empirical model was better compared to the developed GEP model. Moreover, the authors opine that the superiority of the AI technique is not specific, however, strongly depends on the nature (non-linearity) of the problem. One AI technique may perform better for a particular problem, whereas; some other model may surpass it in accuracy for a different situation. In continuation of previous research by Murad et al. [26] for developing a GEP tree-based model for flexural strength of an FRP-reinforced concrete beam, it is desirable to evaluate the performance of other models such as DT and GBT in solving a similar problem. Therefore, this research concentrates on developing novel DT and GBT models for estimating the flexural capacity of FRP reinforced concrete beams and comparing the capabilities of empirical relations and AI solution in terms of correlations and error analysis.

## 2. Methodolgy

### 2.1. Experimental Database

**Figure 1:**
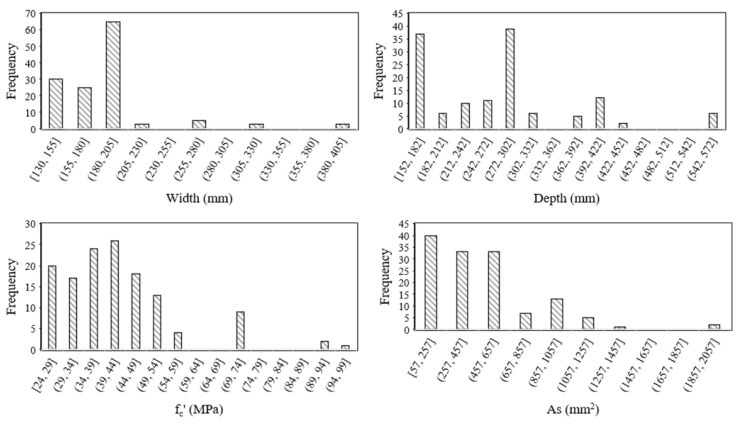

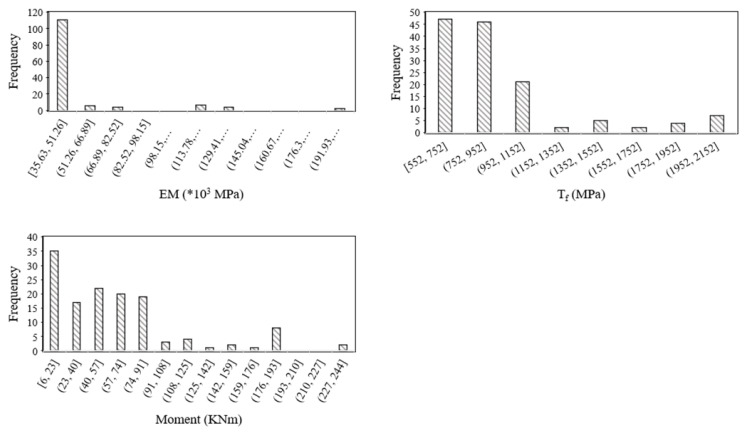
Distribution histograms of the variables (*W*, *D*, *f_c_′*, *As*, *EM*, *T_f_*, *M*) used in the development of models.

The experimental database was collected from various experimental studies listed in Table 1. It can be seen that flexural strength is considered a function of six input parameters, namely; width of beam (*W*), depth of beam (*D*), compressive strength of concrete (*f_c_′*), area of flexural reinforcement (*A_s_*), Elastic modulus of FRP rebar (*EM*), and the tensile strength of rebar at failure (*T_f_*) as expressed in Equation (1).

(1) $$M=f\;\left(\;W,\;D,\;f_{c}^{\prime },\;A_{s}.\;EM,\;T_{f}\right)$$

where, *M* is flexural capacity. The role of these input parameters in contributing towards bending capacity is evident from the well-known ACI formulations expressed as Equation (2) through (6). The distribution histogram and descriptive statistics of the variables used in the study are shown in Figure 1 and Table 2. The histograms show that the majority of specimens (almost 80%) tested in different experimental studies comprises a width range of 130–205 mm, depth range of 152–302 mm, *f_c_′* of 24–54 MPa, *A_s_* of 57–657 mm^2^, *EM* of 35,630–51,260 MPa, and *T_f_* of 552–1152 MPa (Table 2). The standard deviation values reflected from Table 2 shows that the models are developed from a broader range of variable values.

### 2.2. Machine Learning Approaches

#### 2.2.1. Decision Tree

A Decision tree (DT) belongs to a family of supervised machine learning algorithms. As its name suggests, DT is similar to a tree having any branches which represent a possible reaction or different outcomes to a problem. DTs have the ability to visualize all the probable outcomes to a problem under all circumstances and thus have great importance in decision making [75]. DTs provide a better interpretation of model outputs compared to “black-box” models such as neural nets [76]. Due to their simple and easy-to-understand analytics and their precision on multiple data forms, DTs have found many applications in various fields [77,78].

Each tree consists of nodes and branches in the DT model. Each node denotes the features in a classification category, while every subset describes a possible value that the node can take [79]. The instance space from each internal node is divided into two or multiple sub-spaces in accordance with a specific discrete function of input attributes values. At the model output, each leaf is assigned to a unique category that is representative of the most fitting target value. The values in the leaves of DTs are also known as weights in some literature. DTs are sequential models that rationally combine the outcome of simple tests where each one compares a nominal attribute against a set of possible values or a numeric attribute versus a threshold value [80]. The partitioning scheme in DTs is designed to determine the possible partition values along the midpoint of a set of consecutive unique responses for each feature (i.e., gene expression). Different scoring criteria as Gini index (*Gini*), Information Gain (*InfoGain*) are used to compare and evaluate each of the possible tree partition values (Equations (1)–(4)) [75].

(2) $$InfoGain=Info\left(Parent\right)-\sum_{k}\left(p_{k}\right)Info\left(Child_{k}\right)$$

(3) $$Info\left(q\right)=-\sum_{j}\left(\frac{N_{j}\left(t\right)}{N\left(t\right)}\right)){\;\log}_{2}\left(\frac{N_{j}\left(t\right)}{N\left(t\right)}\right)$$

(4) $$Gini=impurity\left(Parent\right)-\sum_{k}\left(p_{k}\right)impurity\left(Child_{k}\right)$$

(5) $$impurity=1-\sum_{j}{\left\|\frac{p\left(j\right)N_{j}\left(t\right)}{N_{j}}\right\|}^{2}$$

where $N_{\left(t\right)}\;$ is the number of samples in node $t;\;N_{j}$ represents the number of samples belonging to the class  j; $N_{j}\left(t\right)$ is the class j samples associated with node t.  The terms $q\;\mathrm{and}\;p_{k}$ denotes the features sub-space and portion of samples passed to kth sub-space, respectively.

#### 2.2.2. Gradient Boosting Tree

The fundamental intuitive idea behind all boosting algorithms is to combine multiple weak learners that should result in classification and regression models with improved predictive performance compared to a single model [81]. In machine learning, the weak learner refers to a model that performs slightly better than a random chance. This concept, which is known as “strength of weak learnability”, was originated from the introduction of Ababoost in the early 1990s [82]. For gradient boosting trees, such weak learners are shallow decision trees.

Three main elements are involved during the predictive modeling of gradient boosting trees, i.e., (i) a loss function to be optimized, (ii) a weak learner for making predictions, (iii) and an additive model for combining the weak learners in order to minimize the loss function. The adopted loss function is typically influenced by the type of problem or task being addressed. For regression problems, a squared loss is the conventional choice, whereas, for classification tasks, an exponential error is a plausible loss function [81]. However, there may be circumstances in where other loss functions such as binomial deviance, Huber loss or absolute error could be more appropriate. The weak learners (decision trees) are constructed in a greedy manner by choosing the best split points (based on nodes purity index such as *Gini*) to minimize the loss. When adding the trees, a stochastic gradient descent procedure is employed to optimize the loss. Predictions of each tree in the gradient boosting tree algorithm are added sequentially, and the overall prediction error decreases as the iteration progresses. It is important that weak learners remain weak despite having some skill. An efficient heuristic is one having constrained tree creation which can be undertaken using several constraints such as number of trees, tree depth, number of nodes or leaves, observations per split, and minimum improvement to loss. In addition to their structure, additional constraints may be imposed to improve their performance. The leaf weight values can be optimized using different regularization functions which are intended to overcome the “overfitting” problem by placing restrictions on model parameters [83]. In the context of GBT, this refers to controlling the iterations (*T*) or trees during the training process. Additive models of GBT with potentially all input variables (*x*) may be represented by the general expression shown in Equation (6), while the function for optimizing the number of iterations is shown in Equation (7).

(6) $$f\left(x\right)=\sum_{t=1}^{T}B_{t}h\left(x;a_{t}\right)$$

(7) $$f_{t}\left(x\right)=f_{t-1}(x)+\tau \;.\;\beta_{t}h\left(x;a_{t}\right)$$

where the term $h\left(x;a_{t}\right)$ is often regarded as a simple function characterized by the multiplier β and set of parameters (a = {a1, a2,…}).The parameter has a retarding effect on the learning rate of the series, which means that for achieving better accuracy, the series has to be long enough to compensate for shrinkage.

#### 2.2.3. Development of the Model and Hyper-Parameters Tuning

**Figure 2:**
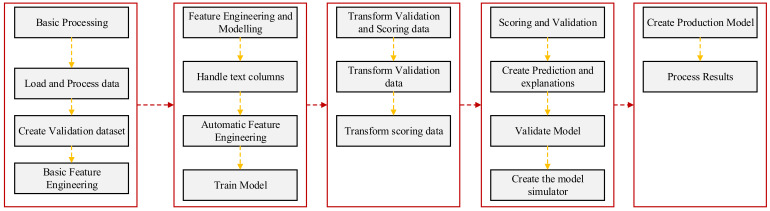
Development of DT and GBT models.

Two models, namely DT and GBT were created in a rapidminer environment following the methodology explained in Figure 2. Overall, the modelling encompasses basic processing, feature modelling, transform validation and scoring data, validation of the models alongside generating scores, weights and simulator, production of the model, and finally delivering the results.

Firstly, basic processing of the database was initiated. The database was subjected to pre-processing. A single row of all the inputs was created to be deployed into the model, attributes were converted into nominal or real values, attributes were labelled, the target variable was defined, and input variables were selected based on their correlations with the target variable. Subsequently, the data were partitioned into the training (60%) and validation datasets (40%). Some basic features were introduced, such as handling unknown values, replacing missing values, and remembering known and unknown values, which were then subjected to feature engineering and modelling. This includes operators, such as handling the columns with the text, automating feature engineering, optimization, cross-validation operator for introducing the specific ML model, applying the ML model, and training the model based on the optimized hyperparameters. After this, the transformation of the training and scoring data (no known target value) to missing values was completed. The models were applied to the validation and scoring data to validate the models. A model simulator was created for future predictions of the new data. Finally, a production model was generated based on the training and validation sets using the same hyperparameters to deliver the results.

The hyperparameters of the DT and GBT were tuned using the trial and error method listed in Table 3. For the DT model, maximal depth was varied from 2 to 25, whereas the minimal error rate was obtained for 7. Similarly, for the GBT model, the number of trees, maximal depth and learning rate were varied from 30–150, 2–7, and 0.001–0.1, respectively. The optimum results were obtained for 90 trees, 2 maximal depth and a 0.1 learning rate.

#### 2.2.4. Evaluation Criteria

The models were evaluated using correlation coefficient (*R*), mean absolute error (*MAE*), and root mean square error (*RMSE*) in accordance with the previous literature [84]. The mathematical equations for these statistical evaluation functions are shown as Equations (8)–(10), respectively.

(8) $$R=\frac{\sum_{i=1}^{n}\left(e_{i}-{\bar{e}}_{i\;}\right)\left(m_{i}-{\bar{m}}_{i\;}\right)}{\sqrt{\sum_{i=1}^{n}{\left(e_{i}-{\bar{e}}_{i\;}\right)}^{2}{\left(m_{i}-{\bar{m}}_{i\;}\right)}^{2}}}$$

(9) $$MAE=\frac{\sum_{i=1}^{n}|e_{i}-m_{i}|}{n}$$

(10) $$RMSE=\sqrt{\frac{\sum_{i=1}^{n}{\left(e_{i}-m_{i\;}\right)}^{2}}{n}}$$

where *e_i_* and *m_i_* are *n*th experimental and model results, respectively; $\bar{e_{i\;}}\;$ and $\bar{m_{i}}$ denote the average values of experimental and model results, respectively, and n is the number of samples in the data set.

## 3. Results and Discussions

### 3.1. Pearson’s Linear Correlations

**Figure 3:**
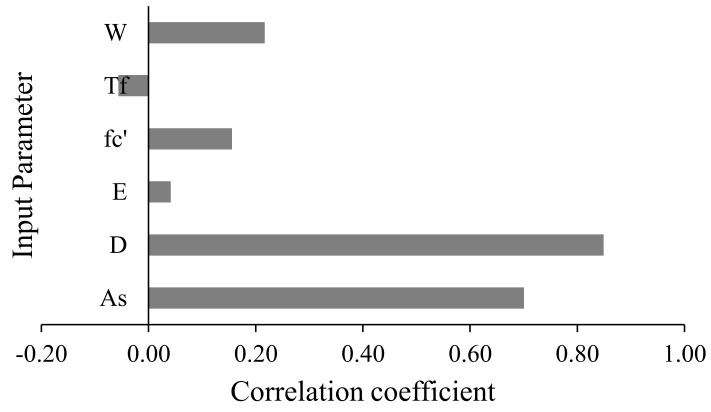
Correlation strength between input and output variables.

In order to evaluate the correlation among the variables, the data employed in the current study were investigated for linear Pearson’s correlation. It can be seen that *A_s_*, and *D* have a strong positive correlation with the flexural capacity of beams. The basic ACI equation for flexural strength of beam also inhibits a similar trend in flexural capacity with an increase in *A_s_* and *D*. The width of the beam (*W*) and *f_c_′* show comparatively moderate positive correlation with *M*. The remaining attributes *EM* and *T_f_* have minor linear correlations, suggesting the existence of non-linear correlations between the inputs and the target variable. The detailed coefficient matrix provided can be seen from Table 4 and Figure 3.

### 3.2. Prediction Performance of the Developed Models

**Figure 4:**
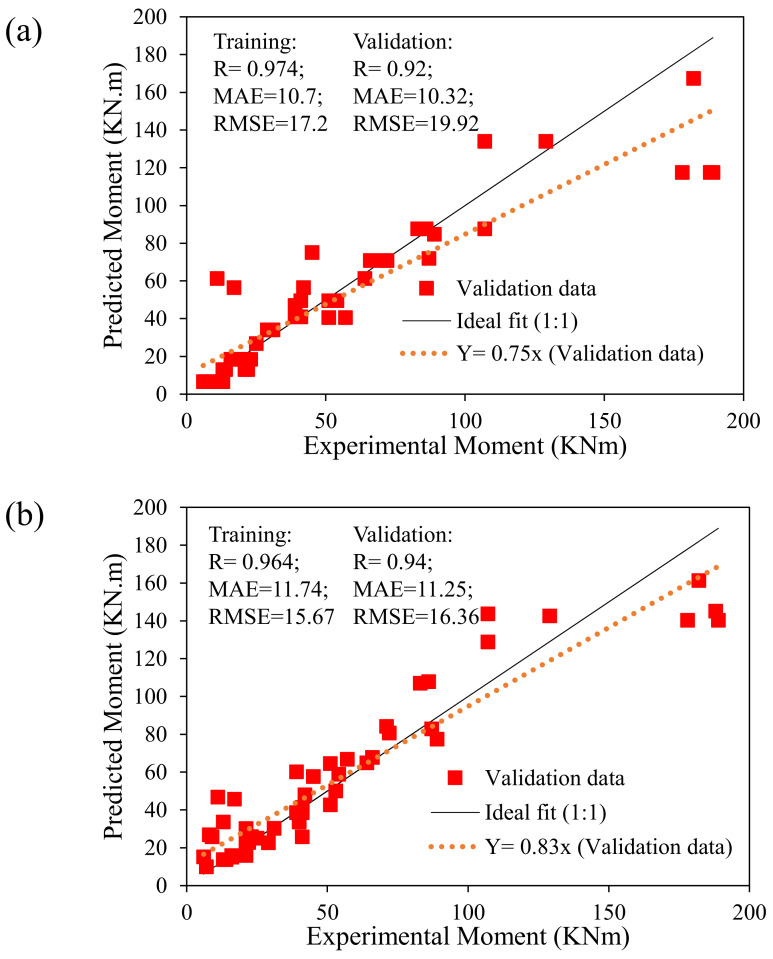
Prediction performance of the developed model: (**a**) DT, and (**b**) GBT.

This section concentrates on statistical evaluation of the developed models in terms of correlation coefficient, MAE, RMSE and the slope of regression line to assess the robustness, effectiveness, and comparative investigation of the developed DT and GBT models for estimating the flexural strength of FRP-reinforced concrete beams. For the development of a reliable and efficient AI model, the ratio between the number of observed records (i.e., 60% training and 40% validation data points which in this case are 80 and 54, respectively) and contributing input variable (six in the current study) must not be less than three and preferably more than 5 [85]. In this research study, for considered flexural strength of FRP-reinforced concrete beams, this ratio equals 13.33 in the training and 9 in the validation phase, which is far beyond the recommended limit, representing relatively more reliable development of AI models. The observed (experimental) and forecasted moments for FRP beams (Figure 4a) for the DT model (Figure 4b) and for the GBT model in the training and validation stage are visualized along with the performance indicator (i.e., slope, R, MAE, and RMSE). The 45-degree standard regression line represents the ideal fitted line with a slope exactly equaling 1. The distribution of the plotted points must be nearer to the standard line, with a slope greater than 0.8, minimal error metrics (MAE and RMSE) and R > 0.8 for reliable performance and strongly correlated models [36,86]. The slope of the regression line for DT and GBT corresponds to 0.75 and 0.83, respectively. In the case of the GBT model, the slope is greater than 0.8, hence representing a more robust performance compared to the DT model. The values of R for the training data of DT and GBT models are 0.974 and 0.964, respectively. Similarly, the validation data also yielded comparable R values equaling 0.92 and 0.94 for DT and GBT models, respectively. The magnitude of R in both the training and validation suggests strongly correlated models; however, R in the case of GBT is greater in the validation phase for the GBT model. Moreover, the values of R in the validation phase are almost comparable to that of the training phase, hence ruling out the problems of overfitting in the developed models.

It is noteworthy that a higher value of R is not the sole indicator to judge the robustness of the AI model. Therefore, several error indices such as MAE and RMSE were also considered in this study. Mainly, while training the model, the optimization process targets the minimization of MAE alongside higher correlation magnitudes. The values of MAE was observed as 10.7 MPa and 11.74 MPa for the training phase of DT and GBT models, respectively. The validation dataset yielded 10.31 and 11.25 MPa for DT and GBT models. RMSE equaled 17.2 MPa and 15.67 MPa in the training stage and 19.92 MPa and 16.36 MPa in the validation stage, respectively, for DT and GBT models.

Based on the statistical evaluation from the validation phase, the value of R and the slope of the regression line were greater in the GBT model. For the same data, the value of RMSE was smaller for the GBT model; hence, the GBT model can be considered more robust compared to DT model.

**Figure 5:**
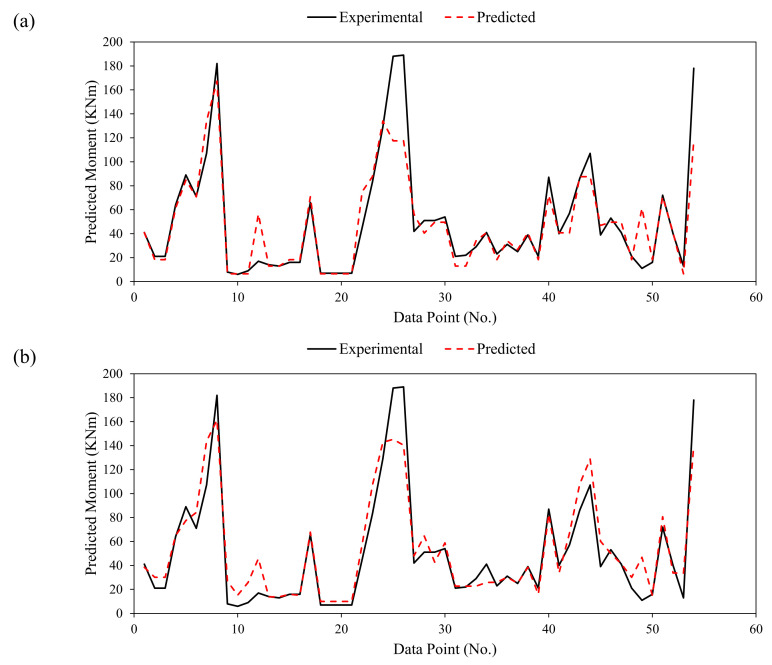
Tracing of experimental values by the predictions in the validation phase of the (**a**) DT, and (**b**) GBT models.

**Figure 6:**
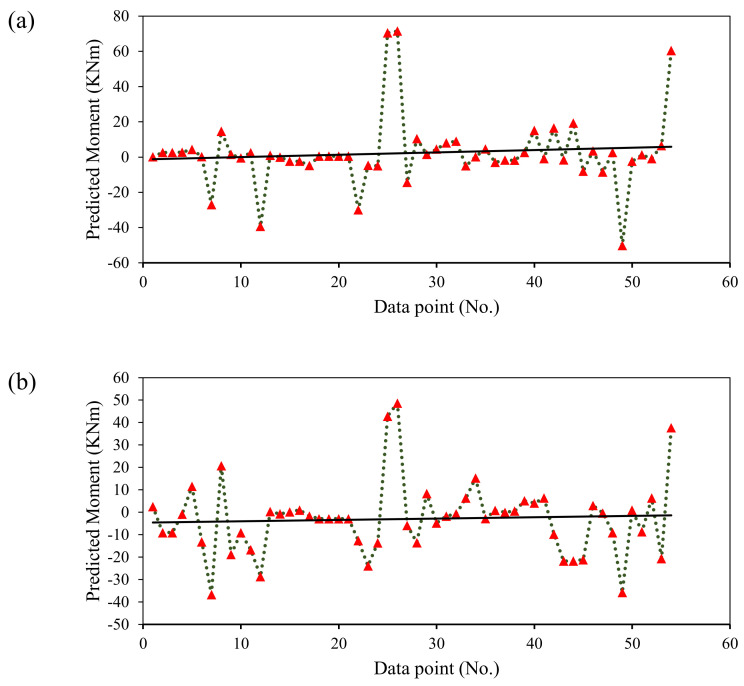
Error Analysis of the proposed models in the validation phase: (**a**) DT, and (**b**) GBT.

It is imperative to evaluate the overall performance of the models, examining the error between experimental records and model-predicted output [87,88]. It can be observed from Figure 5 that predictions followed the experimental results more closely for both the developed models. Figure 6 displays the error generated in the validation stage for DT and GBT models. It can be seen that for DT and GBT models, the maximum of the points were within ±10 MPa. The value of MAE for the test data in the case of DT model was 10.32 MPa, whereas, for the GBT model it was 11.25 MPa as shown in Figure 4. Therefore, the results from statistical viewpoint and relative error (Figure 6) show that the error in the developed models was within 20% (10.32/62.4 = 16.5% and 11.25/62.4 = 18.02%), where 62.4 is average value of the flexural strength of the database used in this study. The trend line of the errors intersects the y-axis at zero, reflecting the minimal errors in the developed models. Thus, in addition to higher correlation and lower error statistics, the developed models can be effectively used to estimate the flexural strength of FRP-reinforced concrete beams that will aid designers and practitioners to avoid heavy testing and save money and time.

### 3.3. Second Level Validation of the Models (Parametric and Sensitivity Analysis)

For this purpose, a simulated data set was established such that the input variable was varied uniformly between its extremes, and the other input variables were kept constant at their average values, as shown in Table 5. The change in the target variable was plotted against the variable input to obtain the parametric influence of the particular variable. Similarly, a simulated dataset was employed for sensitivity analysis. The difference in the values of the target variable with respect to each input variable was normalized to obtain the relative percentage of each contributing variable. Parametric and sensitivity analyses were conducted using the GBT model due to its high accuracy in comparison to DT model.

**Figure 7:**
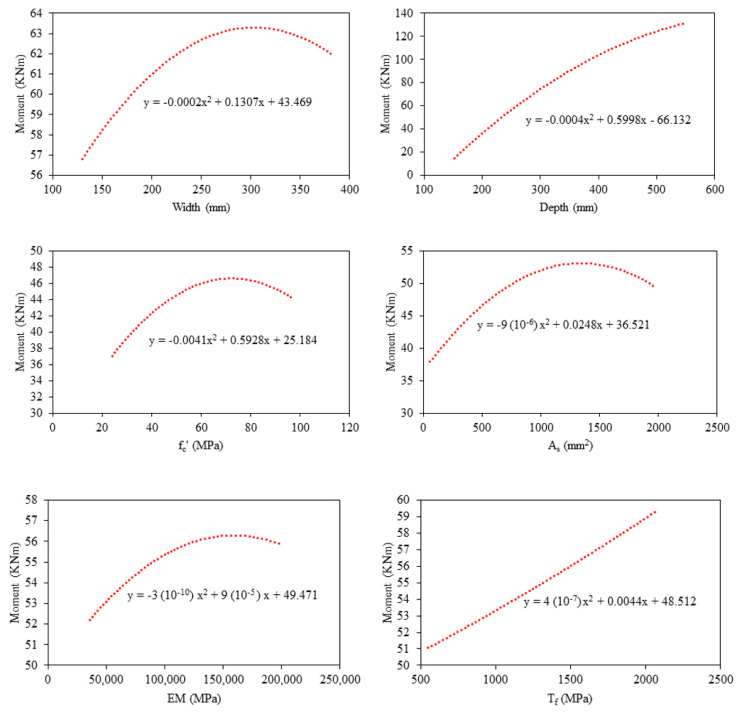
Parametric analysis of the input variable (*W*, *D*, *f_c_′*, *As*, *EM*, *T_f_*) against moment (M) using GBT Model.

**Figure 8:**
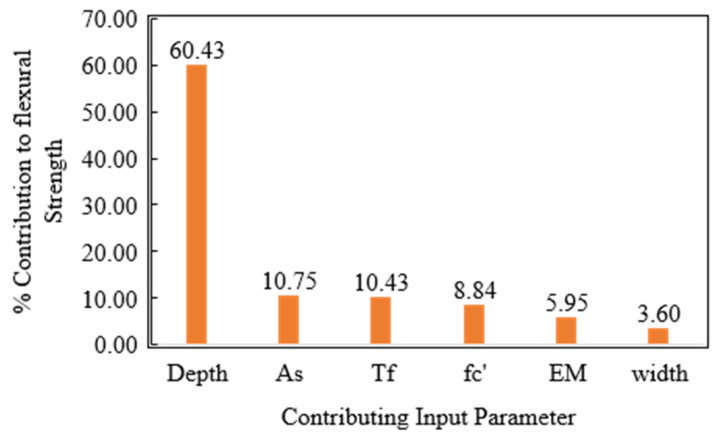
Percentage contribution of input variables using GBT model.

Figure 7 presents the parametric study based on the GBT model. The increase in width from 130–381 mm yielded a change of almost 6 kN-m in the bending capacity. The change in the depth significantly affected the bending capacity, almost 120 kN-m, with the increase in depth from 152 to 550 mm. Bending Moment also increased with increasing *fc′*, *A_s_*, *EM* and *T_f_* as shown in Figure 7. It can be observed that moment capacity significantly changed with the variation in the depth rather than the width of the specimen. The empirical equations and principles of mechanics also suggest similar variations in bending moment with respect to the change in the depth of the beam. Moreover, the increase was not significant with a rise in elastic modulus of FRP rebar. The sensitivity analysis showed that depth of the beam contributed 60% of bending capacity, followed by *A_s_*, *T_f_*, *fc′*, *EM* and *W*, respectively, as shown in Figure 8. The parametric and sensitivity analysis results are in line with the principles of mechanics and empirical equations, suggesting the reliability of the developed model.

### 3.4. Comparison with Previously Developed Models and ACI

**Figure 9:**
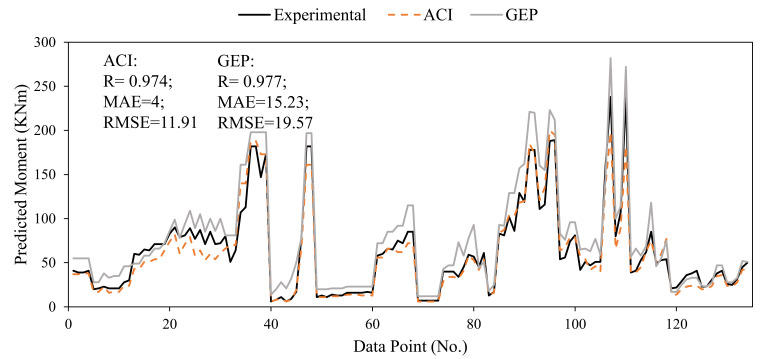
Comparison of ACI and existing AI models.

The accuracy of the developed model was compared with that of already available formulations for flexural strength calculation of FRP-reinforced concrete beams as per ACI 440-17 and previously developed AI models. The equations given as Equation (11) through (15) were employed to calculate the flexural capacity of FRP-reinforced concrete beams. The prediction made by Murad et al. [26] using GEP are reported in Figure 9 for comparison. The statistical evaluation of Figure 9 demonstrates that the GEP model had a higher correlation compared to ACI model of 0.977 and 0.974, respectively. The other indices were MAE (4, 11.91) and RMSE (15.23, 19.27) for the ACI and GEP models, respectively. In contrast to the correlation values, the magnitude of errors was higher in the case of the GEP model compared to the ACI formulation. For statistical evaluation, the magnitude of errors should be small alongside correlation coefficients for an accurate model. Hence, it is inferred that R should not be solely used as an evaluation index for the developed model. In the comparison of GEP and ACI models, ACI was more accurate because of comparable correlation and least error values. Figure 9 also shows that ACI follows the experimental values more closely compared to the GEP model.

Comparing the developed GBT model with the existing models, it can be seen that the GBT model has an almost comparable R of 0.964 to that of the ACI and GEP model. The magnitude of MAE and RMSE were 11.25 and 16.36, respectively. The magnitude of errors was less than in the previously developed GEP model; however, the existing ACI formulations surpassed the accuracy of the developed GBT model.

(11) $$\rho_{f}=\frac{A_{f}}{bd}$$

$\rho_{f}\;$ is FRP reinforcement ratio, $A_{f}$ is the area of longitudinal flexural reinforcement (mm^2^), *b* is width of the beam (mm), and *d* is the depth of the beam (mm).

(12) $$\beta_{1}=\left\{\begin{array}{l} 17\leq f_{c}^{\prime }\leq 28\;\;\;\;\;\;\;\;\;\;\;\;\;\;\;\;\;\;\;\;\beta_{1}=0.85\;\;\\ 28<f_{c}^{\prime }<55\;\;\;\;\;\beta_{1}=0.85-\frac{0.05\left(f_{c}^{\prime }-28\right)}{7}\\ f_{c}^{\prime }\geq 55\;\;\;\;\;\;\;\;\;\;\;\;\;\;\;\;\;\;\;\;\;\;\;\;\;\;\;\;\;\;\beta_{1}=0.65 \end{array}\right.$$

$\beta_{1}$ is compressive stress block parameter, $f_{c}^{\prime }$ is the concrete compressive strength

When $\rho_{f}>\rho_{b}$

(13) $$f_{f}=\sqrt{\frac{{\left(E_{f}\varepsilon_{cu}\right)}^{2}}{4}+\frac{0.85\beta_{1}f_{c}^{\prime }}{\rho_{f}}E_{f}\varepsilon_{cu}-0.5E_{f}\varepsilon_{cu}\leq f_{fu}}$$

$\rho_{b}\;$ is a balanced reinforcement ratio. *f_f_* is tensile stress of FRP rebar at failure, $E_{f}$ is elastic modulus of longitudal FRP bars, $\varepsilon_{cu}$ is ultimate concrete strain = 0.003, $f_{fu}$ is ultimate tensile strength of FRP rabars

When $\rho_{f}<\rho_{b}$

(14) $$c_{b}=\left(\frac{\varepsilon_{cu}}{\varepsilon_{cu}+\varepsilon_{fut}}\right)d$$

(15) $$M_{n}=A_{f}f_{fu}\left(d-\frac{\beta_{1}c_{b}}{2}\right)\;$$

*c* is the distance from extreme compression fiber to the neutral axis of the member (mm), and *c_b_* is the distance from extreme compression fiber to the neutral axis of the member at balanced strain condition (mm).

## 4. Conclusions

This article presents estimation of the bending capacity of FRP-reinforced concrete beams. Previously, Murad et al. [26] developed a GEP tree-based model for flexural capacity of concrete beams reinforced with FRP rebars. It was shown that the strength achieved from the existing ACI equations for flexural capacity deviated from the experimental results. Therefore, the GEP model was found to have better accuracy than ACI 440 in terms of correlations (R) only. Upon other statistical evaluations of their results, our research demonstrated a higher error matrix of GEP predictions compared to ACI equations. Therefore, this research presented modern regression AI techniques namely DT and GBT, for this purpose. The following major conclusions were drawn from this study.

1. The value of the correlation coefficient (R) for DT and GBT models were significantly higher than 0.8 (0.974 and 0.964 for the training stage and 0.92 and 0.94 for the validation stage, respectively), reflecting a solid agreement of input attributes in predicting flexural strength. Error evaluation such as MAE (10.32 kN-m) showed lower values in the validation phase in the case of DT models, whereas lower RMSE (16.36 kN-m) in the GBT model was observed. The performance of both the models were comparable; however, based on the comparison of the slope of validation data recorded as 0.83 (more closer to 1) for GBT models against 0.75 for the DT model and higher R for the validation phase, the GBT model can be considered more accurate and robust.
2. The parametric study revealed a similar trend of the target variables with the change in the input variables coherent with the literature, further validating the trained model. The sensitivity analysis revealed the depth of the beam as the most influential parameter contributing towards flexural strength.
3. The currently developed GBT model surpassed the accuracy of the previously developed GEP model. Hence, the GBT model can effectively predict flexural strength; however, the existing ACI equations are more reliable than the current and previously developed AI models. While comparing the models, it was shown that R should not be used as a single parameter in assessing the performance of the AI models; rather, a few error indices, specifically the MAE should be included.

## Figures and Tables

**Table 1:** Details of input and output parameters used in development of the model.

Flexural Capacity (kN-m) Target Varaible	Number of Specimens	Input Parameters						
		Depth (mm)	Width (mm)	Compressive Strength (*f_c_^′^*) MPa	Flexural Reinforcemnet (*A_s_*) mm^2^	Elastic Modulus (*EM*) MPa	Tensile Strength Rebar at Failure (*T_f_*) MPa	References
20–30	8	180	130	46–97	238–475	38,000	773	[54]
39–41	4	240	200	35–36	508	43,370	885	[55]
71–90	12	300	200	39–41	254–1013	40,000–122,000	617–1988	[56]
49–66	6	300	180	35	253–507	40,000	695	[57]
81–198	9	300–550	200	43–52	573	42,000–49,000	641–689	[58]
80–182	3	300–550	43	573	600	45,000	600	[59]
6–17	14	200–300	150	28–50	57–113	38,000	650	[60]
11–17	12	152–203	191–381	28	80–320	41,400	830	[61]
6–34	9	152–250	150–152	25–36	71–429	45,000–44,800	760–1000	[62]
58–85	8	300	200	45–52	349–1046	37,600	773	[63]
34–57	4	210–300	200	31–41	507–1134	35,630–43,370	700–886	[64]
52–54	2	300	200	24–27	88–226	200,000	1061–2000	[65]
14–16	2	152	152	49–52	63–99	140,000	1900	[66]
81–189	12	400	200	29–73	261–1162	48,700–69,300	762–1639	[67]
42–81	6	305	152	29–45	355–1013	45,500–50,600	552–896	[68]
47–51	3	229	178	48	219–1077	41,000–124,000	552–896	[69]
80–238	5	380	280	34–43	339–1964	38,000–40,200	582–603	[70]
39–85	5	270–294	200	42–54	299–1356	38,000–49,000	552–773	[71]
49–54	3	254–256	230	40	226–603	50,000	1000	[72]
21–41	6	165	180	30	115–424	42,900–46,600	1075–1121	[73]
23–50	10	165	180	47–70	171–636	42,900–130,000	1029–2068	[74]

**Table 2:** Descriptive statistics of input and output variables.

Parameters	Minimum	Maximum	Mean	Median	Standard Deviation	Skewness	Kurtosis
Input parameters							
Width, W (mm)	130	381	194.2	200	3.9	2	6
Depth, D (mm)	152	550	274.4	294	8.6	0.9	0.7
Concrete compressive strength, *fc′* (MPa)	24	97	42.9	41	1.2	1.6	3.4
Bottom tensile reinforcement, As (mm^2^)	57	1964	482.9	425	30.8	1.5	3.2
Elastic Modulus, EM (MPa)	35,630	200,000	53,060	43,370	2550	3	10
Tensile strength at failure (*T_f_*)	552	2069	927.6	773	33.2	1.7	2.2
Flexural capacity, M (kN-m)	6	238	62.4	51	4.3	1.5	2

**Table 3:** Tuning hyper-parameters of DT and GBT models.

Model	Parameter	Value	Error Rate Optimization (%)
DT	Maximal depth	2	32.3
		4	21.8
		**7**	**20.0**
		10	20.0
		15	20.0
		25	20.0
GBT	Number of trees, maximum depth, Learning rate	30, 2, 0.001	37.3
		90, 2, 0.001	36.1
		150, 2, 0.001	35.2
		30, 4, 0.001	37.3
		90, 4, 0.001	36.1
		150, 4, 0.001	35.1
		30, 7, 0.001	37.3
		90, 7, 0.001	36.1
		150, 7, 0.001	35.1
		30, 2, 0.01	33.3
		90, 2, 0.01	27.3
		150, 2, 0.01	23.3
		30, 4, 0.01	33.1
		90, 4, 0.01	26.9
		150, 4, 0.01	23.1
		30, 7, 0.01	33.1
		90, 7, 0.01	26.9
		150, 7, 0.01	23.1
		30, 2, 0.1	18.2
		**90, 2, 0.1**	**17.5**
		150, 2, 0.1	17.5
		30, 4, 0.1	17.5
		90, 4, 0.1	18.1
		150, 4, 0.1	18.3
		30, 7, 0.1	17.5
		90, 7, 0.1	18.1
		150, 7, 0.1	18.3

**Table 4:** Correlation matrix among variables used in the development of models.

Attribute	*A_s_*	*D*	*EM*	*fc′*	*T_f_*	*M*	*W*
*A_s_*	1.00	0.44	−0.17	0.09	−0.23	0.70	0.09
*D*	0.44	1.00	0.01	0.03	−0.17	0.85	0.19
*EM*	−0.17	0.01	1.00	−0.02	0.76	0.04	−0.04
*fc′*	0.09	0.03	−0.02	1.00	0.06	0.16	−0.31
*T_f_*	−0.23	−0.17	0.76	0.06	1.00	−0.06	−0.04
*M*	0.70	0.85	0.04	0.16	−0.06	1.00	0.22
*W*	0.09	0.19	−0.04	−0.31	−0.04	0.22	1.00

**Table 5:** Simulated dataset for parametric and sensitivity analysis.

Variable Input Parameters		No. of Datapoints	Constant Input Parameters
Parameter	Range		
Width (mm)	130–381	20	Depth = 274.40 mm, *fc′* = 42.85 MPa, *As* = 482.85 (mm^2^), *EM* = 53,060 MPa, *Tf* = 927.59 MPa
Depth (mm)	152–550	20	Width = 194.25 mm, *fc′* = 42.85 MPa, *As* = 482.85 (mm^2^), *EM* = 53,060 MPa, *Tf* = 927.59 MPa
*f_c_′* (MPa)	24–97	20	Depth = 274.40 mm, Width = 194.25 mm, *As* = 482.85 (mm^2^), *EM* = 53,060 MPa, *Tf* = 927.59 MPa
*As* (mm^2^)	57–1964	20	Depth = 274.40 mm, Width = 194.25 mm, *fc′* = 42.85 MPa, *EM* = 53,060 MPa, *Tf* = 927.59 MPa
EM (MPa)	35,630–200,000	20	Depth = 274.40 mm, Width = 194.25 mm, *fc′* = 42.85 MPa, As = 482.85 (mm^2^), *Tf* = 927.59 MPa
*T_f_* (MPa)	552–2069	20	Depth = 274.40 mm, Width = 194.25 mm, *fc′* = 42.85 MPa, As = 482.85 (mm^2^), EM = 53,060 MPa

## Data Availability

The data used in this research has been properly cited and reported in the main text.

## References

[1] Van Damme H. (2018). Concrete material science: Past, present, and future innovations. Cem. Concr. Res..

[2] Bart F., Cau-di-Coumes C., Frizon F., Lorente S. (2012). Cement-Based Materials for Nuclear Waste Storage.

[3] Broomfield J. (2003). Corrosion of Steel in Concrete: Understanding, Investigation and Repair.

[4] Rodrigues R., Gaboreau S., Gance J., Ignatiadis I., Betelu S. (2021). Reinforced concrete structures: A review of corrosion mechanisms and advances in electrical methods for corrosion monitoring. Constr. Build. Mater..

[5] Böhni H. (2005). Corrosion in Reinforced Concrete Structures.

[6] Ahmad S. (2003). Reinforcement corrosion in concrete structures, its monitoring and service life prediction—A review. Cem. Concr. Compos..

[7] Polder R.B., Peelen W.H.A., Courage W.M.G. (2012). Non-traditional assessment and maintenance methods for aging concrete structures—Technical and non-technical issues. Mater. Corros..

[8] Angst U.M. (2018). Challenges and opportunities in corrosion of steel in concrete. Mater. Struct..

[9] Xiao J., Qiang C., Nanni A., Zhang K. (2017). Use of sea-sand and seawater in concrete construction: Current status and future opportunities. Constr. Build. Mater..

[10] Iqbal M., Zhao Q., Zhang D., Jalal F.E., Jamal A. (2021). Evaluation of tensile strength degradation of gfrp rebars in harsh alkaline conditions using non-linear genetic-based models. Mater. Struct..

[11] Benmokrane B., Manalo A., Bouhet J.-C., Mohamed K., Robert M. (2017). Effects of diameter on the durability of glass-fiber-reinforced-polymer (gfrp) bars conditioned in alkaline solution. J. Compo. Constr..

[12] Mousavi T., Shafei E. (2019). Impact response of hybrid frp-steel reinforced concrete slabs. Presented at Structures.

[13] Hassan A., Khairallah F., Mamdouh H., Kamal M. (2018). Evaluation of self-compacting concrete columns reinforced with steel and frp bars with different strengthening techniques. Presented at Structures.

[14] Li Y., Wang Y., Ou J. (2014). Mechanical behavior of bfrp-steel composite plate under axial tension. Polymers.

[15] Iqbal M., Zhang D., Jalal F.E. (2021). Durability evaluation of gfrp rebars in harsh alkaline environment using optimized tree-based random forest model. J. Ocean Eng. Sci..

[16] Bencardino F., Condello A., Ombres L. (2016). Numerical and analytical modeling of concrete beams with steel, frp and hybrid frp-steel reinforcements. Compos. Struct..

[17] Zhou Y., Zheng Y., Pan J., Sui L., Xing F., Sun H., Li P. (2019). Experimental investigations on corrosion resistance of innovative steel-frp composite bars using X-ray microcomputed tomography. Compos. Part B Eng..

[18] Zhou J., Chen X., Chen S. (2011). Durability and service life prediction of gfrp bars embedded in concrete under acid environment. Nucl. Eng. Des..

[19] Wang L., Mao Y., Lv H., Chen S., Li W. (2018). Bond properties between frp bars and coral concrete under seawater conditions at 30, 60, and 80 °C. Constr. Build. Mater..

[20] Li S., Guo S., Yao Y., Jin Z., Shi C., Zhu D. (2021). The effects of aging in seawater and SWSSC and strain rate on the tensile performance of gfrp/bfrp composites: A critical review. Constr. Build. Mater..

[21] Jin Q., Chen P., Gao Y., Du A., Liu D., Sun L. (2020). Tensile strength and degradation of gfrp bars under combined effects of mechanical load and alkaline solution. Materials.

[22] Chen Y., Davalos J.F., Ray I., Kim H.-Y. (2007). Accelerated aging tests for evaluations of durability performance of frp reinforcing bars for concrete structures. Compos. Struct..

[23] Chen Y., Davalos J.F., Ray I. (2006). Durability prediction for gfrp reinforcing bars using short-term data of accelerated aging tests. J. Compos. Constr..

[24] Daelemans L., Van Paepegem W., D’Hooge D.R., De Clerck K. (2018). Excellent nanofiber adhesion for hybrid polymer materials with high toughness based on matrix interdiffusion during chemical conversion. Adv. Funct. Mater..

[25] Nanni A. (2005). Guide for the design and construction of concrete reinforced with frp bars (ACI 440.1R-03). Structures Congress 2005.

[26] Murad Y., Tarawneh A., Arar F., Al-Zu’Bi A., Al-Ghwairi A., Al-Jaafreh A., Tarawneh M. (2021). Flexural strength prediction for concrete beams reinforced with FRP bars using gene expression programming. Structures.

[27] Joshani M., Koloor S., Abdullah R. (2012). Damage Mechanics Model for Fracture Process of Steel-Concrete Composite Slabs. Appl. Mech. Mater..

[28] Lammens N., Luyckx G., VAN Paepegem W., Degrieck J. (2016). Finite element prediction of resin pocket geometries around arbitrary inclusions in composites: Case study for an embedded optical fiber interrogator. Compos. Struct..

[29] Sevenois R.D.B., VAN Paepegem W. (2015). Fatigue damage modeling techniques for textile composites: Review and comparison with unidirectional composite modeling techniques. Appl. Mech. Rev..

[30] Trong D.K., Pham B.T., Jalal F.E., Iqbal M., Roussis P.C., Mamou A., Ferentinou M., Vu D.Q., Dam N.D., Tran Q.A. (2021). On random subspace optimization-based hybrid computing models predicting the california bearing ratio of soils. Materials.

[31] Tran T.-H., Dam N.D., Jalal F.E., Al-Ansari N., Ho L.S., Van Phong T., Iqbal M., Van Le H., Nguyen H.B.T., Prakash I. (2021). GIS-based soft computing models for landslide susceptibility mapping: A case study of pithoragarh district, uttarakhand state, India. Math. Probl. Eng..

[32] Jamal A., Al-Ahmadi H.M., Butt F.M., Iqbal M., Almoshaogeh M., Ali S. (2021). Metaheuristics for Traffic Control and Optimization: Current Challenges and Prospects. https://www.intechopen.com/online-first/78022.

[33] Jalal F.E., Xu Y., Li X., Jamhiri B., Iqbal M. (2021). Fractal approach in expansive clay-based materials with special focus on compacted gmz bentonite in nuclear waste disposal: A systematic review. Environ. Sci. Pollut. Res..

[34] Iqbal M., Onyelowe K.C., Jalal F.E. (2021). Smart computing models of california bearing ratio, unconfined compressive strength, and resistance value of activated ash-modified soft clay soil with adaptive neuro-fuzzy inference system and ensemble random forest regression techniques. Multiscale Multidiscip. Model. Exp. Des..

[35] Azimi-Pour M., Eskandari-Naddaf H. (2018). ANN and GEP prediction for simultaneous effect of nano and micro silica on the compressive and flexural strength of cement mortar. Constr. Build. Mater..

[36] Khan M.A., Zafar A., Akbar A., Javed M., Mosavi A. (2021). Application of gene expression programming (GEP) for the prediction of compressive strength of geopolymer concrete. Materials.

[37] Aslam F., Elkotb M.A., Iqtidar A., Khan M.A., Javed M.F., Usanova K.I., Alamri S., Musarat M.A. (2021). Compressive strength prediction of rice husk ash using multiphysics genetic expression programming. Ain Shams Eng. J..

[38] Faradonbeh R.S., Hasanipanah M., Amnieh H.B., Armaghani D.J., Monjezi M. (2018). Development of gp and gep models to estimate an environmental issue induced by blasting operation. Environ. Monit. Assess..

[39] Awoyera P.O., Kirgiz M.S., Viloria A., Ovallos-Gazabon D. (2020). Estimating strength properties of geopolymer self-compacting concrete using machine learning techniques. J. Mater. Res. Technol..

[40] Wang X.-Y. (2020). Prediction of flexural strength of natural pozzolana and limestone blended concrete using machine learning based models. Presented at IOP Conference Series: Materials Science and Engineering.

[41] Ahmad A., Ostrowski K., Maślak M., Farooq F., Mehmood I., Nafees A. (2021). Comparative study of supervised machine learning algorithms for predicting the compressive strength of concrete at high temperature. Materials.

[42] Congro M., Monteiro V.M.D.A., Brandão A.L., dos Santos B.F., Roehl D., Silva F.D.A. (2021). Prediction of the residual flexural strength of fiber reinforced concrete using artificial neural networks. Constr. Build. Mater..

[43] Lee S., Lee C. (2014). Prediction of shear strength of frp-reinforced concrete flexural members without stirrups using artificial neural networks. Eng. Struct..

[44] Köroğlu M.A. (2018). Artificial neural network for predicting the flexural bond strength of frp bars in concrete. Sci. Eng. Compos. Mater..

[45] Lee S., Moy S. (2007). A Method for predicting the flexural strength of RC beams strengthened with carbon fiber reinforced polymer. J. Reinf. Plast. Compos..

[46] Yang J.-M., Min K.-H., Shin H.-O., Yoon Y.-S. (2012). Effect of steel and synthetic fibers on flexural behavior of high-strength concrete beams reinforced with frp bars. Compos. Part B Eng..

[47] Pham H., Al-Mahaidi R. (2004). Assessment of available prediction models for the strength of FRP retrofitted RC beams. Compos. Struct..

[48] Ghosh A., Maiti R. (2021). Soil erosion susceptibility assessment using logistic regression, decision tree and random forest: Study on the mayurakshi river basin of eastern india. Environ. Earth Sci..

[49] Maloney K.O., Weller D.E., Russell M.J., Hothorn T. (2009). Classifying the biological condition of small streams: An example using benthic macroinvertebrates. J. N. Am. Benthol. Soc..

[50] Zounemat-Kermani M., Rajaee T., Ramezani-Charmahineh A., Adamowski J.F. (2017). Estimating the aeration coefficient and air demand in bottom outlet conduits of dams using GEP and decision tree methods. Flow Meas. Instrum..

[51] Song H., Ahmad A., Farooq F., Ostrowski K.A., Maślak M., Czarnecki S., Aslam F. (2021). Predicting the compressive strength of concrete with fly ash admixture using machine learning algorithms. Constr. Build. Mater..

[52] Kamari A., Pournik M., Rostami A., Amirlatifi A., Mohammadi A.H. (2017). Characterizing the CO_2_-brine interfacial tension (ift) using robust modeling approaches: A comparative study. J. Mol. Liq..

[53] Huat C.Y., Moosavi S.M.H., Mohammed A.S., Armaghani D.J., Ulrikh D.V., Monjezi M., Lai S.H. (2021). Factors influencing pile friction bearing capacity: Proposing a novel procedure based on gradient boosted tree technique. Sustainability.

[54] Thériault M., Benmokrane B. (1998). Effects of frp reinforcement ratio and concrete strength on flexural behavior of concrete beams. J. Compos. Constr..

[55] Almusallam T., Al-Salloum Y., Alsayed S., Amjad M. Behavior of concrete beams doubly reinforced by frp bars. Proceedings of the Third International Symposium on Non-Metallic (FRP) Reinforcement for Concrete Structures (FRPRCS-3).

[56] Kassem C., Farghaly A.S., Benmokrane B. (2011). Evaluation of flexural behavior and serviceability performance of concrete beams reinforced with frp bars. J. Compos. Constr..

[57] Toutanji H.A., Saafi M. (2000). Flexural behavior of concrete beams reinforced with glass fiber-reinforced polymer (gfrp) bars. ACI Struct. J..

[58] Benmokrane B., Chaallal O., Masmoudi R. (1995). Glass fibre reinforced plastic (gfrp) rebars for concrete structures. Constr. Build. Mater..

[59] Benmokrane B., Masmoudi R. (1996). Flexural response of concrete beams reinforced with frp reinforcing bars. Struct. J..

[60] Ashour A. (2006). Flexural and shear capacities of concrete beams reinforced with GFRP bars. Constr. Build. Mater..

[61] Yost J.R., Goodspeed C.H., Schmeckpeper E.R. (2001). Flexural performance of concrete beams reinforced with frp grids. J. Compos. Constr..

[62] Brown V.L., Bartholomew C.L. (1993). Frp reinforcing bars in reinforced concrete members. Mater. J..

[63] Masmoudi R., Theriault M., Benmokrane B. (1998). Flexural behavior of concrete beams reinforced with deformed fiber reinforced plastic reinforcing rods. Struct. J..

[64] Duranovic N., Pilakoutas K., Waldron P. (1997). Tests on concrete beams reinforced with glass fibre reinforced plastic bars. Non-metallic (FRP) Reinf. Concr. Struct..

[65] Alsayed S., Al-Salloum Y., Almusallam T. (2000). Performance of glass fiber reinforced plastic bars as a reinforcing material for concrete structures. Compos. Part B Eng..

[66] Bischoff P.H. (2005). Reevaluation of deflection prediction for concrete beams reinforced with steel and fiber reinforced polymer bars. J. Struct. Eng..

[67] El-Nemr B.A., Ahmed E.A., Benmokrane B. (2013). Flexural behavior and serviceability of normal- and high-strength concrete beams reinforced with glass fiber-reinforced polymer bars. ACI Struct. J..

[68] Faza S.S. (1991). Bending and Bond Behavior and Design of Concrete Beams Reinforced with Fiber-Reinforced Plastic Rebars.

[69] Wang H., Belarbi A. (2005). Flexural behavior of fiber-reinforced-concrete beams reinforced with frp rebars. ACI Struct. J..

[70] Lau D., Pam H.J. (2010). Experimental study of hybrid frp reinforced concrete beams. Eng. Struct..

[71] Gao D., Benmokrane B. (2001). Calculation method of flexural capacity of gfrp-reinforced concrete beam. J. Hydraul. Eng..

[72] El Refai A., Abed F., Al-Rahmani A. (2015). Structural performance and serviceability of concrete beams reinforced with hybrid (gfrp and steel) bars. Constr. Build. Mater..

[73] Alkhraisha H., Mhanna H., Tello N., Abed F. (2020). Serviceability and flexural behavior of concrete beams reinforced with basalt fiber-reinforced polymer (bfrp) bars exposed to harsh conditions. Polymers.

[74] Abed F., Al-Mimar M., Ahmed S. (2021). Performance of bfrp rc beams using high strength concrete. Compos. Part C Open Access.

[75] Myles A.J., Feudale R.N., Liu Y., Woody N.A., Brown S.D. (2004). An introduction to decision tree modeling. J. Chemom..

[76] Ijaz M., Lan L., Zahid M., Jamal A. (2021). A comparative study of machine learning classifiers for injury severity prediction of crashes involving three-wheeled motorized rickshaw. Accid. Anal. Prev..

[77] Jamal A., Zahid M., Rahman M.T., Al-Ahmadi H.M., Almoshaogeh M., Farooq D., Ahmad M. (2021). Injury severity prediction of traffic crashes with ensemble machine learning techniques: A comparative study. Int. J. Inj. Control Saf. Promot..

[78] Ullah I., Liu K., Yamamoto T., Al Mamlook R.E., Jamal A. (2021). A comparative performance of machine learning algorithm to predict electric vehicles energy consumption: A path towards sustainability. Energy Environ..

[79] Mrva J., Neupauer S., Hudec L., Sevcech J., Kapec P. Decision Support in Medical Data Using 3D Decision Tree Visualisation. Proceedings of the 2019 E-Health and Bioengineering Conference (EHB).

[80] Kotsiantis S.B. (2011). Decision trees: A recent overview. Artif. Intell. Rev..

[81] Guelman L. (2012). Gradient boosting trees for auto insurance loss cost modeling and prediction. Expert Syst. Appl..

[82] Sun Y., Kamel M.S., Wong A.K., Wang Y. (2007). Cost-sensitive boosting for classification of imbalanced data. Pattern Recognit..

[83] Cortes C., Mohri M., Storcheus D. (2019). Regularized gradient boosting. Adv. Neural Inf. Process. Syst..

[84] Iqbal M.F., Liu Q.-F., Azim I., Zhu X., Yang J., Javed M.F., Rauf M. (2020). Prediction of mechanical properties of green concrete incorporating waste foundry sand based on gene expression programming. J. Hazard. Mater..

[85] Iqbal M.F., Javed M.F., Rauf M., Azim I., Ashraf M., Yang J., Liu Q.-F. (2021). Sustainable utilization of foundry waste: Forecasting mechanical properties of foundry sand based concrete using multi-expression programming. Sci. Total Environ..

[86] Azim I., Yang J., Iqbal M.F., Javed M.F., Nazar S., Wang F., Liu Q.-F. (2020). Semi-analytical model for compressive arch action capacity of RC frame structures. Structures.

[87] Babanajad S.K., Gandomi A.H., Alavi A.H. (2017). New prediction models for concrete ultimate strength under true-triaxial stress states: An evolutionary approach. Adv. Eng. Softw..

[88] Alavi A.H., Gandomi A.H., Nejad H.C., Mollahasani A., Rashed A. (2013). Design equations for prediction of pressuremeter soil deformation moduli utilizing expression programming systems. Neural Comput. Appl..

